# Association of *GSTM1* and *GSTT1* Null Genotypes with Toluene Diisocyanate-Induced Asthma

**DOI:** 10.1155/2022/7977937

**Published:** 2022-02-11

**Authors:** Jong-Uk Lee, Ji-Yeon Jeong, Min Kyung Kim, Sun A. Min, Jong-Sook Park, Choon-Sik Park

**Affiliations:** ^1^Division of Allergy and Respiratory Medicine, Department of Internal Medicine, Soonchunhyang University Bucheon Hospital, Bucheon, Republic of Korea; ^2^Department of Interdisciplinary Program in Biomedical Science Major, Soonchuhyang University, Asan, Republic of Korea; ^3^PulmoBioPark Co., Ltd., Soonchunhyang University Bucheon Hospital, Bucheon, Republic of Korea

## Abstract

**Background:**

Toluene diisocyanate (TDI) causes occupational asthma by generating oxidative stress, leading to tissue injury and inflammation. Glutathione transferases (GSTs) are detoxifying enzymes that eliminate oxidative stress. We examined whether the genotypes of the *GSTM1* and *GSTT1* genes are associated with TDI-induced occupational asthma (TDI-OA).

**Methods:**

The study population consisted of 26 asthmatics with a positive response to the TDI challenge (TDI-PA) and 27 asthmatics with negative responses (TDI-NA). *GSTM1* and *GSTT1* null and wild-type genotypes were determined using multiplex PCR. The plasma GSTM1 and GSTT1 protein concentrations were determined using ELISA.

**Results:**

The *GSTM1* null genotype was more frequent in the TDI-PA than in the TDI-NA (77.8 *vs*. 50.0%, OR = 3.5, *p*=0.03), while the frequency of the *GSTT1* null genotype tended to be higher in the TDI-PA than in the TDI-NA (59.3 *vs*. 42.3%, OR = 1.98, *p*=0.21). When analyzed together, the *GSTM1*/*GSTT1* null genotype was more frequent in the TDI-PA than in the TDI-NA (48.2 *vs*. 15.3%, OR = 6.5, *p*=0.04). The decline in the FEV in 1 s after TDI challenge was higher with the *GSTM1*/*GSTT1* null than the *GSTM1* wild-type/*GSTT1* null genotypes (24.29% *vs*. 7.47%, *p*=0.02). The plasma GSTM1 level was lower with the *GSTM1* null than with the *GSTM1* wild-type genotypes both before (13.7 *vs*. 16.6 ng/mg, *p*=0.04) and after (12.9 *vs*. 17.1 ng/mg, *p*=0.007) the TDI challenge, while the GSTT1 level was not changed with either the *GSTT1* null or wild-type genotype.

**Conclusions:**

The *GSTM1* null genotype, but not *GSTT1* alone, may confer susceptibility to TDI-OA. However, the genetic effect of the *GSTM1* null genotype may be enhanced synergistically by the *GSTT1* null genotype. The genetic effect of *GSTM1* was validated in the plasma as the GSTM1 protein level. Therefore, the *GSTM1* and *GSTT1* genotypes may be useful diagnostic markers for TDI-OA.

## 1. Introduction

Highly reactive toluene diisocyanate (TDI), diphenyl-methane diisocyanate, and hexamethylene diisocyanate are widely used globally for manufacturing polyurethane foams, paints, and lacquers and cause occupational asthma (OA) [1] accounting for more than half of the OA cases in Korea [2]. TDI-induced OA (TDI-OA) is characterized by airway hyper-responsiveness, inflammation, and remodeling [3, 4]. Several immunological and nonimmunological mechanisms are thought to be involved in the pathogenesis of TDI-OA, as in other types of OA [5, 6]. One nonimmunological mechanism involves physical interactions between diisocyanate and airway epithelium that induce the production of proinflammatory cytokines and chemokines, which recruit inflammatory cells [7]. Isocyanates also induce oxidative stress directly in inflammatory monocyte cell lines [8], lymphocytes [9], neutrophils [10], constitutional epithelial [11], and endothelial [12] cell lines. These processes are accompanied by redox imbalances that overwhelm antioxidant systems in the airways [13] as well as systemically [14]. Several complex antioxidant systems protect cells from oxidative stress, such as glutathione S-transferases (GSTs), thioredoxin peroxidases, catalases, glutathione reductases, and superoxide dismutases [15].

GSTs are a family of eukaryotic and prokaryotic phase II metabolic isozymes that catalyze the conjugation of the reduced form of glutathione to xenobiotic substrates for detoxification [16]. The GST family includes cytosolic, mitochondrial, and microsomal proteins [17]. Human cytosolic GSTs can be divided into five main classes: alpha, mu, pi, theta, and zeta [18]. The human *GSTM* (GST*μ*) gene family on chromosome 1p13.3 consists of five different isotypes, *GSTM1* to *GSTM5* [19], and the theta class (*GSTT*) on chr22 includes *GSTT1*, *GSTT2*, and *GSTT2B* [20]. The null genotypes result in no GST function, and therefore the conjugation reaction and subsequent elimination of the toxic products of oxidative stress are inefficient, which causes inflammatory and immune dysfunction in chronic disorders. *GSTM1* mutations are associated with risks of inflammatory bowel diseases [21], type 2 diabetes mellitus [22], and atherosclerotic cardiovascular diseases [23]. *GSTM1* and *GSTT1* null genotypes are also associated with risks of chronic obstructive lung disease [24] and lung cancer [25].

A recent meta-analysis demonstrated that *GSTM1* and *GSTT1* null genotypes are associated with an increased risk of asthma [26]. TDI-OA may develop more frequently in individuals with null genotypes than in those with wild-type genotype via enhanced oxidative stress in the airways and whole body[14]. Isocyanate-induced asthma is associated with the *GSTM1* null genotype in Caucasians [27], and the *GSTP1* Val/Val genotype is less frequent in asthmatics exposed to TDI for 10 or more years [28]. Thus, individuals with the null genotypes for both *GSTM1* and *GSTT1* may be susceptible to TDI-OA in a synergistic manner. However, few studies have examined the association between the *GSTM1*/*GSTT1* null genotypes and TDI-OA. Furthermore, most studies diagnosed TDI-OA based on a history of work exposure to the causative agent and the presence of asthma symptoms. However, this approach is not always satisfactory for diagnosing OA [29]. Thus, in the present study, TDI-OA was diagnosed by specific inhalation challenge (SIC) using TDI solution because SIC is considered the standard reference test [30]. Then, the distributions of deletion mutants of *GSTM1* and *GSTT1* were compared between TDI-challenge-positive (TDI-PA) and -negative (TDI-NA) asthma patients to explore the possible associations between the null genotypes and the risk for TDI-OA. Concomitantly, the plasma GSTM1 and GSTT1 protein concentrations were analyzed to validate the genetic effects.

## 2. Materials and Methods

### 2.1. Study Subjects

Buffy coats and plasma (*n* = 53) were obtained from a biobank at Soonchunhyang University Hospital, Bucheon (schbc-biobank-2019-009-01), Korea, after the study was approved by the Ethics Committee of Soonchunhyang University (IRB No. 201905-BR-020-01). Informed written consent for study participation and sample donation was obtained from each subject. Additionally, this study used fully anonymized data collected previously as part of the biobank at Soonchunhyang University Bucheon Hospital.

All subjects had a history of work exposure and asthma symptoms; however, 27 subjects had positive (TDI-PA group) and 26 negative (TDI-NA group) responses to SIC with TDI, as described previously [31]. The decline in the forced expiratory volume in 1 s (FEV1) after the TDI challenge (dFEV1-TDI) was calculated as ((FEV1 before challenge – the lowest FEV1 after provocation)/FEV1 before challenge) × 100 (%). Atopy was determined by a positive skin test to at least one of the following common inhalant allergens: house dust mites, tree, grass, weed pollens, animal dander, and *Alternaria* (Bencard, Brentford, UK).

### 2.2. Isolation of DNA and Genotyping of GSTM1 and GSTT1

**Figure 1:**
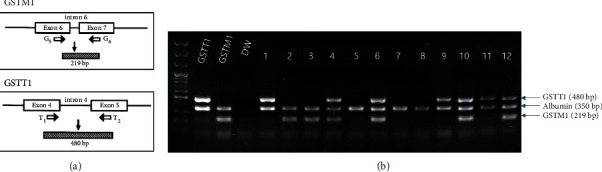
Primer positions (a) and gel electrophoresis of GSTM1 and GSTT1 RT-PCR products and albumin (b). Lanes 1, 9, and 11: *GSTT1* wild/*GSTM1* null genotype; lanes 5, 7, and 8: *GSTT1*/*GSTM1* null genotype; lanes 4, 6, 10, and 12: *GSTT1*/*GSTM1* wild genotype; and lanes 2 and 3, *GSTT1* null/*GSTM1* wild genotype.

DNA was isolated using the QIAGEN DNA isolation kit following the manufacturer's protocol (QIAGEN, Hilden, Germany). The DNA concentration and quality were measured using the NanoDrop instrument (NanoDrop Technologies, Wilmington, DE, USA) and by agarose gel electrophoresis. The *GSTM1* and *GSTT1* genotypes were determined simultaneously in a single multiplex PCR assay (Figure 1(a)). Briefly, isolated DNA (20 ng) was amplified in a 25 *μ*L reaction mixture containing 10 pmol GSTM1 (5′-GAACTCCCTGAAAAGCTAAAGC and 5′-GTTGGGCTCAAATATACGGTGG) and GSTT1 (5′-TICCTTACTGGTCCTCACATCTC and 5′-TCACCGGATCATGGCCAGCA) primers. As an internal control, albumin was coamplified using the primers 5′-GCCCTCTGCTAACAAGTCCTAC and 5′-GCCCTAAAAAGAAAATCGCCAATC. PCR was performed using the Multiplex PCR assay kit (Takara Bio, Shiga, Japan) under the following protocol: 5 min at 94°C, followed by 30 cycles at 94°C for 1 min, 63°C for 1 min, and 72°C for 1 min. The PCR products were analyzed electrophoretically on ethidium bromide-stained 1.5% agarose gels. The absence of *GSTM1* or *GSTT1* PCR products was defined as the null genotype (Figure 1(b)).

### 2.3. Measuring Plasma GSTM1 and GSTT1 Protein Concentrations

The plasma GSTM1 and GSTT1 concentrations were measured using an ELISA kit (MyBioSource, San Diego, CA, USA) according to the manufacturer's instructions. A microtiter plate was precoated with antibodies specific for GSTM1 and GSTT1. The absorbance at 450 nm was determined by an ELISA reader, and the GSTM1 and GSTT1 protein concentrations were determined by the optical density of the samples in comparison with a standard curve. The total protein concentration was determined by the BCA assay using bovine serum albumin as a standard.

### 2.4. Statistical Analysis

The data were analyzed using IBM SPSS ver. 20.0. (IBM, Armonk, NY, USA). The Pearson *χ*^2^ test was used to compare the differences in the *GSTM1* and *GSTT1* genotype frequencies between TDI-PA and TDI-NA. The relative risks of the genotypes for TDI-PA were analyzed using logistic regression and presented using odd ratios (ORs) with 95% confidence intervals (CIs). The GSTM1 and GSTT1 protein concentrations were compared between TDI-PA and TDI-NA according to the *GST* genotype using the nonparametric Mann–Whitney *U*-test and the Wilcoxon signed rank test after testing for normality using the Shapiro–Wilk test. Values of *p* < 0.05 were considered significant.

## 3. Results

### 3.1. Comparing GSTM1 and GSTT1 Null Genotypes between the TDI-PA and TDI-NA Groups

Of the 53 asthmatics, 27 were TDI-PA (51%) and 26 were TDI-NA (49%). The TDI-PA group had a significantly greater FEV1 decline after the TDI challenge and a higher serum total IgE level compared with those of the TDI-NA group (*p* < 0.05) (Table 1).  Table 2 shows the distributions of *GSTM1* and *GSTT1* null genotypes. The respective frequencies of the *GSTM1* null and wild-type genotypes were 77.8% and 22.2% in the TDI-PA group (*n* = 27) and 50.0% and 50.0% in the TDI-NA group (*n* = 26) (Table 2). The proportion of participants with the null genotype was significantly higher in the TDI-PA group (OR: 3.5, 95% CI: 1.06–11.49, *p*=0.035) (Table 2). The respective frequencies of the *GSTT1* null and wild-type genotypes were 59.3% and 40.7% in the TDI-PA group and 42.3% and 57.7% in the TDI-NA group (Table 2). The proportion of participants with the null genotype tended to be higher, but not significantly, in the TDI-PA group (*p*=0.217).

### 3.2. Combined Effect of GSTM1 and GSTT1 Null Genotypes on the TDI-Induced Response

The study subjects were divided into four groups according to the wild-type and null genotypes of both *GSTM1* and *GSTT1*: (1) *GSTM1*/*GSTT1* wild; (2) *GSTM1* null/*GSTT1* wild; (3) *GSTM1* wild/*GSTT1* null; and (4) *GSTM1*/*GSTT1* null. In the TDI-PA group (*n* = 27), *GSTM1*/*GSTT1* null was the most frequent genotype (*n* = 13, 48.2%), followed by *GSTM1* null/*GSTT1* wild (*n* = 8, 29.6%), *GSTM1* wild/*GSTT1* null (*n* = 3, 11.1%), and *GSTM1*/*GSTT1* wild (*n* = 3, 11.1%) (Table 3). In the TDI-NA group (*n* = 26), *GSTM1* null/*GSTT1* wild was the most frequent genotype (*n* = 9, 34.6%), followed by *GSTM1* wild/*GSTT1* null (*n* = 7, 26.9%), *GSTM1*/*GSTT1* wild (*n* = 6, 23.0%), and *GSTM1*/*GSTT1* null (*n* = 4, 15.3%). Accordingly, the *GSTM1*/*GSTT1* null genotype was three times more frequent in the TDI-PA than in the TDI-NA groups (OR = 6.50 (95% CI: 1.09–38.63), *p*=0.04).

**Figure 2:**
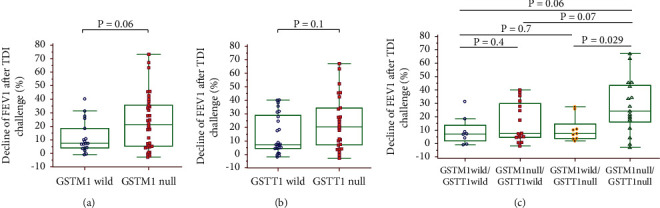
Decline in FEV1 (%) after the TDI challenge according to the *GSTM1* and *GSTT1* genotypes. Comparison of dFEV1-TDI between the *GSTM1* null and wild-type genotypes (a), between the *GSTT1* null and wild-type genotypes (b), and among the combined *GSTM1* and *GSTT1* genotypes (c).

The dFEV1-TDI ((pre – post FEV1)/pre FEV1 × 100)) was evaluated according to the *GSTM1* and *GSTT1* genotypes: dFEV1-TDI tended to be higher with the *GSTM1* null than wild-type genotypes (21.17% *vs*. 7.38%, *p*=0.06) (Figure 2(a)) and with the *GSTT1* null than wild-type genotypes (20.39% *vs*. 7.45%, *p*=0.1) (Figure 2(b)). In a combined analysis of the *GSTM1* and *GSTT1* genotypes, dFEV1-TDI was highest with the *GSTM1*/*GSTT1* null type among the four genotypes, with a significant difference compared with the *GSTM1* wild/*GSTT1* null genotype (24.29% *vs*. 7.47%, *p*=0.029) (Figure 2(c)).

### 3.3. GSTM1 and GSTT1 Protein Levels in Plasma according to Genotype

**Figure 3:**
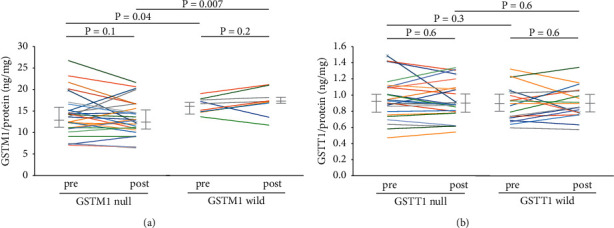
Plasma GSTM1 and GSTT1 protein levels according to genotype. The plasma GSTM1 protein concentrations before and after the TDI challenge in individuals with the null (*n* = 31) or wild-type (*n* = 12) *GSTM1* genotype (a). The plasma GSTT1 protein concentrations before and after the TDI challenge in individuals with the null (*n* = 25) or wild-type (*n* = 18) *GSTT1* genotypes (b).

The plasma GSTM1 protein concentrations did not change after the TDI challenge in the null (*n* = 31) or wild-type (*n* = 12) *GSTM1* genotypes (Figure 3(a)). However, the concentrations were significantly lower with the *GSTM1* null than with the wild-type genotype before (13.7 *vs*. 16.1 ng/mg protein, *p*=0.04) and after (12.9 *vs*. 17.1 ng/mg protein, *p*=0.007) (Figure 3(a)) the TDI challenge. The plasma GSTT1 protein concentration did not change after the TDI challenge with the null (*n* = 25) or wild-type (*n* = 18) *GSTT1* genotype (Figure 3(b)). The GSTT1 protein concentration did not differ between *GSTT1* null and wild-type before and after the TDI challenge (Figure 3(b)).

## 4. Discussion

We found that the *GSTM1* null genotype was more frequent in the TDI-PA than in the TDI-NA group and was associated with a three-fold increased risk of developing asthma after TDI exposure. In comparison, the frequency of the *GSTT1* null genotype was increased, but not significantly, in the TDI-PA group. This suggests that the *GSTM1* null genotype, rather than the *GSTT1* null genotype, has a dominant genetic effect on susceptibility to TDI-OA. Because GSTM1 and GSTT1 are involved in conjugation reactions and elimination of toxins to produce oxidative stress and subsequent airway inflammation [32, 33], reduced GST enzymatic function can trigger asthma onset via increased oxidative stress [34]. *GST* polymorphisms modulate the susceptibility to asthma. In a recent meta-analysis of 41 case-control studies, the pooled results showed a significant association of asthma with both the *GSTM1* (OR = 1.21) and *GSTT1* (OR = 1.61) genotypes [26]. Considering these data and ours, the dominant responding *GST* genotypes may depend on the exposure agent.

Genetic effects of the *GST* family on TDI-OA have been reported in several ethnic populations. In a study of Caucasians, the *GSTM1* null genotype was associated with a 1.89-fold higher risk of diisocyanate-induced asthma (*n* = 109) compared with TDI exposure in the absence of asthma (*n* = 73), while the frequency of the *GSTT1* null genotype did not differ between the two groups [27].These findings are in line with our finding that the *GSTM1* null genotype was associated with an approximately three-fold increased risk of TDI-induced asthma, while the *GSTT1* null genotype had an insignificant association. In our study, the frequencies of the *GSTM1* and *GSTT1* null genotypes were 77.8% and 59.3%, respectively, which were greater than those reported in a Caucasian study (54.1% and 11.9%) [27]. This discrepancy might be attributed to racial differences. The *GSTM1* and *GSTT1* null genotype frequencies in the TDI-NA group in our study were 50% and 42.3%, which are similar to those reported in 1,051 Korean males (53.8% and 54.3%, respectively) [35]. A study of workers with SIC-confirmed diisocyanate-induced asthma (*n* = 95) revealed that the *GSTM1* null and *GSTP1* rs762803 genotypes were risk variants [36]. In another study of 92 TDI-OA patients exposed to TDI for 10 or more years, the frequency of the GSTP1 Val/Val genotype was associated with moderate-to-severe airway hyper-responsiveness to methacholine compared with subjects with normal or mild hyper-responsiveness [28]. Thus, of the GST family members, *GSTM1* appears to be most frequently associated with diisocyanate-induced asthma.

Because GSTs have overlapping effects, they act synergistically as antioxidants. Such an effect was most marked for the combination of the *GSTM1* null and *GSTM3* AA genotypes, which was strongly associated with a late reaction in the bronchial provocation test in Caucasians. The genotype combinations *GSTT1* ∗ GSTP1 rs762803 and *GSTM1* ∗ EPHX1 rs2854450 were also associated with diisocyanate-induced asthma [36]. Furthermore, our study first demonstrated that the *GSTM1*/*GSTT1* null genotype conferred a 6.5-fold increased risk of TDI-OA compared with the GSTM1/GSTT1 wild genotype. This suggests a possible additive contribution of *GSTT1* to *GSTM1* null genotypes in the development of TDI-OA. The frequency of homozygous deletion of *GSTM1* and *GSTT1* in our subjects (*n* = 17/53; 32%) was similar to that in a large Korean population (29.1%) and higher than that in a Caucasian population (7.5%) [35]. dFEV1-TDI was highest in subjects with *GSTM1*/*GSTT1* null among the four genotypes, with a significant difference compared with the *GSTM1* wild/*GSTT1* null genotype. FEV1-TDI tended to be higher in subjects with the *GSTM1* null than in wild-type genotypes. This suggests that *GSTM1* and *GSTT1* have a synergistically protective effect.

To validate the genetic effects of *GSTM1* and *GSTT1*, the plasma GSTM1 and GSTT1 protein levels were measured. The GSTM1 protein concentration was significantly downregulated in *GSTM1* null compared with wild-type patients, while the GSTT1 protein concentration did not differ between the genotypes, confirming that *GSTM1* null patients are more susceptible to TDI-OA than *GSTT1* null patients. Interestingly, the plasma GSTM1 and GSTT1 protein concentrations did not change after the TDI challenge regardless of the genotype. There might be delayed induction of these proteins. Because plasma GSTM1 and GSTT1 are produced mainly in the liver [37], a change in hepatic enzyme activity in response to inhalation of TDI might occur later in the circulation. The delayed response of GST proteins has been well demonstrated in diet studies. A 7-day diet of cruciferous vegetables significantly increased the serum GSTM1 concentration [38]. In that study, the GSTM1 protein concentration was significantly higher on day 7 than on day 6 of the diet, suggesting that the response to the diet reached a steady state after 1 week. This may explain why the GSTM1 and GSTT1 levels did not change immediately after the TDI challenge. A long-term workplace exposure study may be needed to evaluate the different *in vivo* responses of GST family members according to genotypes.

Our study has several limitations. First, normal controls and non-TDI-exposed asthmatics were not included. The specificity of *GSTM1* null genotypes to TDI-OA should be analyzed by comparisons with asthmatics with other types of occupational exposure. Second, other isoforms of GSTM and GSTT were not evaluated. Human cytosolic GSTs consist of five main classes (alpha, mu, pi, theta, and zeta) [18], and the human GSTM family consists of five different isotypes (*GSTM1* to *GSTM5*) [19], while the GSTT family includes *GSTT1*, *GSTT2*, and *GSTT2B* [20].These other GST family members compensate for the absence of a functional *GSTM1* enzyme [39]. Finally, we did not have sufficient statistical power to evaluate genotype × TDI interactions. When estimating the sample size for this study, we would have 83.67% power with a sample size of 64. A *post-hoc* calculation based on our results indicates that our power was low (46.53% for overall effects). Therefore, it is possible that the significant results may also be explained by chance. However, our study was strengthened by the enrollment of subjects with correct diagnoses. TDI-OA is often diagnosed based on a history of work exposure to causative agents and the presence of asthma symptoms. However, this approach is not always satisfactory for diagnosing OA.29 Instead, we diagnosed TDI-PA by SIC using a TDI solution, which is considered the standard reference test[30].

## 5. Conclusion

The associations of the genotypes of the *GSTM1* and *GSTT1* genes with TDI-OA were evaluated in 26 TDI-PA and 27 TDI-NA individuals. The *GSTM1* null genotype was more frequent in the TDI-PA group. In the combination analysis, the frequency of the *GSTM1*/*GSTT1* null genotype was higher in the TDI-PA group than in the TDI-NA group. The plasma GSTM1 protein concentration was significantly lower in *GSTM1* null than in wild-type individuals after the TDI challenge, while the GSTT1 protein concentration did not change in *GSTT1* null and wild-type individuals after the TDI challenge. Thus, the *GSTM1* null genotype may cause susceptibility to TDI-PA, and this effect may be synergistically enhanced by the *GSTT1* null genotype. These data suggest that the *GSTM1* and *GSTT1* genotypes are useful diagnostic markers for TDI-OA.

## Figures and Tables

**Table 1:** Clinical characteristics of the study subjects.

Variables	TDI-PA	TDI-NA	*P* value
*N*	27	26	—
Age (years)	58.7 ± 8.6	57.1 ± 10.9	0.60
Sex (male, female)	23/4	21/5	0.72
Smoke (NS/ES/SM)	9/12/6	6/9/11	0.28
Atopy (Y, N)	19/8	15/11	0.39
FVC (% predicted)	87.48 ± 21.31	92.96 ± 12.62	0.26
FEV1 (% predicted)	90.22 ± 24.86	97 ± 13.07	0.22
FEV1_FVC (%)	76.22 ± 16.67	81.42 ± 9.06	0.16
Decline of FEV1 after the TDI challenge (%)	35.15 ± 15.13	4.57 ± 3.98	3.66*E* − 11
Serum total IgE (kU/I)	463.18 ± 689.94	154.23 ± 200.42	0.03

Numeric data are presented as mean ± standard deviation. TDI, toluene diisocyanate; TDI-PA, TDI-positive asthma; TDI-NA, TDI-negative asthma; ES, ex-smokers; NS, never smokers; SM, current smokers. *P* values were obtained using the independent *t*-test or *χ*^2^-test and were considered significant when less than 0.05.

**Table 2:** Comparison of GSTM1 and GSTT1 genotype frequencies according to the response of the TDI challenge.

Genotype	TDI-PA (%)/TDI-NA (%)	OR (95% CI)	*P* value
*GSTM1*			
Wild	6 (22.2)/13 (50.0)	1.0 (reference)	0.035
Null	21 (77.8)/13 (50.0)	3.50 (1.06–11.49)	

*GSTT1*			
Wild	11 (40.7)/15 (57.7)	1.0 (reference)	0.217
Null	16 (59.3)/11 (42.3)	1.98 (0.66–5.91)	

TDI-PA: TDI challenge positive asthma and TDI-NA: TDI challenge negative asthma. *P* values were obtained using logistic regression analysis and were considered significant when less than 0.05.

**Table 3:** Combined analysis of GSTM1 and GSTT1 mutant frequencies according to the response of the TDI challenge.

Genotypes	TDI-PA (%)/TDI-NA (%)	OR (95% CI)	*P* value
*GSTM1 wild/GSTT1 wild*	3 (11.1)/6 (23.0)	1.0 (reference)	—
*GSTM1 null/GSTT1 wild*	8 (29.6)/9 (34.6)	1.77 (0.33–9.55)	0.50
*GSTM1 wild/GSTT1 null*	3 (11.1)/7 (26.9)	0.85 (0.12–5.94)	0.87
*GSTM1 null/GSTT1 null*	13 (48.2)/4 (15.3)	6.50 (1.09–38.63)	0.04

TDI-PA: TDI challenge positive asthma and TDI-NA: TDI challenge negative asthma. *P* values were obtained using logistic regression analysis and were considered significant when less than 0.05.

## Data Availability

The data used to support the findings of this study are available from the corresponding author upon request.

## Abbreviations

TDI: Toluene diisocyanate
OA: Occupational asthma
GSTM1: Glutathione S-transferase Mu 1
GSTT1: Glutathione S-transferase Theta 1.

## Additional Points

(1) The *GSTM1* null genotype may confer susceptibility to TDI-OA. (2) The genetic effect of the *GSTM1* null genotype may be enhanced synergistically by the *GSTT1* null genotype. (3) The *GSTM1* and *GSTT1* genotypes may be useful diagnostic markers for TDI-OA.

## Supplementary Materials

Supplementary Materials
*Study Subjects*. Buffy coats and plasma were obtained from a biobank at Soonchunhyang University Hospital, Bucheon, Korea. Additionally, this study used fully anonymized data collected previously as part of the biobank at Soonchunhyang University Bucheon Hospital.
