# Supplementary material for: Association of GSTM1 and GSTT1 Null Genotypes with Toluene Diisocyanate-Induced Asthma
Source: Can Respir J. 2022 Feb 11;2022:7977937. doi: 10.1155/2022/7977937 (PMC8856815; doi:10.1155/2022/7977937)
Supplement: Supplementary Materials — Study Subjects. Buffy coats and plasma were obtained from a biobank at Soonchunhyang University Hospital, Bucheon, Korea. Additionally, this study used fully anonymized data collected previously as part of the biobank at Soonchunhyang University Bucheon Hospital. [file 7977937.f1.pdf]

SUPPLEMENTARY

MATERIAL METHODS

Study subjects

The study was conducted with the permission of the Ethics Committee of Soonchunhyang University. Buffy coats and plasma were obtained from a biobank at Soonchunhyang University Hospital, Bucheon, Korea, and informed written consent for study participation and sample donation was obtained from each subject. Additionally, this study used fully anonymized data collected previously as part of the biobank at Soonchunhyang University Bucheon Hospital as following the table:

Anonymized ID	Diagnosis	Smoke	Atopy	FVC (% predictied)	FEV1 (% predictied)	FEV1_FVC (%)	Decline of FEV1 after TDI challenge (%)	Serum total IgE (kU/I)	Atopy
BC4061353	TDI-Positive	NS	Y	90	107	93	31.84	331	Y
BC3950614	TDI-positive	NS	N	86	90	16	40.29	320	N
BC3327599	TDI-positive	NS	N	70	75	87	38.27	274	N
BC4066158	TDI-Positive	NS	N	105	115	84	24.29	66.7	N
BC3942544	TDI-Positive	ES	N	99	93	71	45.74	40	N
BC3758267	TDI-positive	ES	Y	87	81	72	27.84	347	Y
BC3794680	TDI-positive	NS	Y	96	111	84	27.83	1282	Y
BC3525833	TDI-positive	SM	Y	95	95	75	17.7	183	Y
BC3463762	TDI-positive	ES	N	92	102	85	25.89	191	N
BC3459727	TDI-positive	ES	Y	85	87	79	17.53	3205	Y
BC3480493	TDI-positive	NS	Y	100	112	89	33.58	120	Y
BC3480172	TDI-positive	NS	Y	75	78	80	31.45	90.3	Y
BC3475840	TDI-positive	ES	Y	95	106	89	73.33	27.2	Y

BC3515750	TDI-positive	NS	N	110	119	83	63.34	264	N
BC3537582	TDI-positive	SM	Y	84	90	81	52.29	216	Y
BC3521948	TDI-positive	NS	Y	96	89	74	20.39	327	Y
BC3550452	TDI-positive	ES	N	76	67	67	42.62	41	N
BC3744205	TDI-positive	ES	N	107	110	80	45.56	150	N
BC3743796	TDI-positive	ES	Y	108	116	83	67.15	45.9	Y
BC3788239	TDI-positive	ES	Y	110	119	79	24.73	268	Y
BC3812893	TDI-Positive	SM	Y	98	102	80	40	635	Y
BC3823324	TDI-positive	ES	Y	73	69	74	27.47	94.7	Y
BC3521816	TDI-positive	SM	Y	72	65	68	18.5	319	Y
BC3941726	TDI-positive	ES	Y	91	87	70	21.96	1331	Y
BC4437715	TDI-positive	SM	Y	82	90	83	19.15	438	Y
BC3796012	TDI-positive	SM	Y	93	88	77	35.67	1706	Y
BC4048896	TDI-positive	ES	Y	73	63	71	34.51	193	Y
BC4059945	TDI-negative	SM	N	61	65	81	5.19	22.8	N
BC3318355	TDI-negative	NS	N	83	83	101	4.31	49	N
BC3741874	TDI-negative	ES	Y	84	92	77	3.63	353	Y
BC3431721	TDI-negative	NS	Y	80	86	86	4.56	24.8	Y
BC3733190	TDI-negative	NS	Y	82	87	83	-0.93	72.8	Y
BC4058343	TDI-negative	SM	Y	102	85	66	-1.82	246	Y
BC3333602	TDI-negative	SM	N	81	94	89	8.96	151	N
BC3690463	TDI-negative	ES	Y	100	99	78	10.16	109	Y
BC3534518	TDI-negative	SM	N	87	96	86	4.2	147	N
BC3321006	TDI-negative	SM	Y	91	101	88	2.02	141	Y
BC3364668	TDI-negative	NS	N	79	95	96	6.87	57.5	N

BC3744141	TDI-negative	SM	Y	103	99	78	7.47	103	Y
BC3748326	TDI-negative	SM	Y	88	89	78	7.24	80.3	Y
BC3473049	TDI-negative	ES	N	88	88	80	0.9	32.5	N
SC4370012	TDI-negative	SM	N	96	91	70	-0.33	999	N
BC3735796	TDI-negative	SM	N	100	115	91	0.63	439	N
BC3603726	TDI-negative	ES	N	105	88	60	10.89	46	N
BC3809095	TDI-negative	ES	Y	100	99	76	11.48	179	Y
BC3474910	TDI-negative	NS	Y	88	100	85	-0.45	66.3	Y
BC3295519	TDI-negative	ES	Y	108	107	75	-2.8	154	Y
BC4326814	TDI-negative	ES	Y	88	92	80	5.86	88.4	Y
BC4315462	TDI-negative	ES	Y	98	102	74	6.96	81.6	Y
BC3414836	TDI-negative	NS	N	125	130	77	7.3	89.3	N
BC4230514	TDI-negative	SM	Y	108	116	85	4.83	17.5	Y
BC4303207	TDI-negative	SM	Y	98	110	83	3.99	26.3	Y
BC3680083	TDI-negative	ES	N	94	113	94	7.61	234	N
